# Active immunization against complement factor C5a: a new therapeutic approach for Alzheimer’s disease

**DOI:** 10.1186/s12974-015-0369-6

**Published:** 2015-08-16

**Authors:** Christine Landlinger, Lisa Oberleitner, Petra Gruber, Birgit Noiges, Kristyna Yatsyk, Radmila Santic, Markus Mandler, Guenther Staffler

**Affiliations:** AFFiRiS AG, Karl-Farkas-Gasse 22, Vienna, 1030 Austria

## Abstract

**Background:**

Alzheimer’s disease (AD) is the most common neurodegenerative disease characterized by neuronal loss due to amyloid beta aggregations, neurofibrillary tangles, and prominent neuroinflammation. Recently, interference with neuroinflammation as a new therapeutic approach for AD treatment gained great interest. Microglia cells, one of the major contributors in neuroinflammation, are activated in response to misfolded proteins such as amyloid β and cell debris leading to a sustained release of pro-inflammatory mediators. Especially, complement factor C5a and its receptor have been found to be up-regulated in microglia in the immediate surroundings of cerebral amyloid plaques and blocking of C5aR resulted in a reduction of pathological markers in a model of AD. Here, we investigate the effect of active vaccination against the complement factor C5a to interfere with neuroinflammation and neuropathologic alterations in a mouse model of AD.

**Methods:**

Short antigenic peptides AFF1 and AFF2, which mimic a C-terminal epitope of C5a, were selected and formulated to vaccines. These vaccines are able to induce a highly specific antibody response to the target protein C5a. Tg2576 mice, a common model of AD, were immunized with these two C5a-peptide vaccines and the induced immune response toward C5a was analyzed by ELISA and Western blot analysis. The influence on memory retention was assessed by a contextual fear conditioning test. Microglia activation and amyloid plaque deposition in the brain was visualized by immunohistochemistry.

**Results:**

Both C5a-targeting vaccines were highly immunogenic and induced sustained antibody titers against C5a. Tg2576 mice vaccinated at early stages of the disease showed significantly improved contextual memory accompanied by the reduction of microglia activation in the hippocampus and cerebral amyloid plaque load compared to control mice. Late-stage immunization also showed a decrease in the number of activated microglia, and improved memory function, however, had no influence on the amyloid β load.

**Conclusion:**

C5a-peptide vaccines represent a safe and well-tolerated immunotherapy, which is able to induce a strong and specific immune response against the pro-inflammatory molecule C5a. In a mouse model of AD, C5a-peptide vaccines reduce microglia activation and thus neuroinflammation, which is supposed to lead to reduced neuronal dysfunction and AD symptomatic decline.

## Background

Alzheimer’s disease (AD) is the most common cause of dementia in the elderly characterized by memory decline and cognitive dysfunction. Approximately 36 million individuals are currently affected worldwide (WHO 2014). The main neuropathological hallmarks in AD are extracellular amyloid β plaques (reviewed in [1]), intracellular neurofibrillary tangles (reviewed in [2]), prominent inflammatory processes (reviewed in [3]), and as a consequence neuron loss. Originally, the deposition of amyloid β peptides was considered to be the most crucial step that ultimately leads to AD dementia described as amyloid cascade hypothesis [4]. Several therapeutics that targeted amyloid β aggregation were tested in clinical trials, however, none of them showed consistent improvements in AD patients (reviewed in [5, 6]). Although amyloid aggregation is still considered to be a driving factor in the onset of AD, it is known that amyloid depositions can occur among the elderly also without cognitive impairment [7, 8]. In addition, it was shown in transgenic mouse models that Aβ alone is not sufficient for cellular and cognitive loss [9].

Besides amyloid β aggregation, a strong activation of inflammatory processes was observed in the brains of AD-affected individuals [10–12]. Reactive microglia cells were found throughout the cortex and hippocampus of patients with AD and were particularly concentrated in the areas of plaque formation [13, 14]. The interference with neuroinflammation has therefore gained considerable attention as a potential therapeutic approach in recent years [3, 15].

Inflammatory processes in AD are primarily triggered by the up-regulation of the complement system in response to misfolded and aggregated proteins or mislocalized nucleic acids and reactive microglia [10, 16, 17]. Prolonged chronic neuroinflammation is thought to reinforce neuronal cell dysfunction and cell death [18, 19]. Notably, the pro-inflammatory complement factor C5a and its receptor have been found to be up-regulated in microglia in the immediate surroundings of cerebral amyloid plaques in different mouse models of AD [20]. It was shown that the blockage of C5aR by the antagonist PMX205 lead to a therapeutic benefit in a rat model of neurodegeneration [21]. This inhibitor was also tested in Tg2576 and 3xTg mice, two different mouse models of AD, and showed improved memory skills and reduced amyloid plaque formation [22]. Furthermore, it was reported that C5-deficient DAB/2 mice, which carry the human APP transgene, had significantly lower Aβ levels compared to C5-sufficient C57BL/6 mice with the identical transgene [23].

In this study, we investigate the therapeutic effect of the interference with neuroinflammation by an active vaccination directed against the complement factor C5a in a mouse model of AD.

For this propose, two antigenic peptides, which are able to induce a humoral immune response against a C-terminal epitope of the murine C5a molecule, were selected and subsequently tested in wild-type (wt) and Tg2576 mice. Both peptide vaccines were able to specifically target C5a and found to ameliorate memory deficits and neuropathology in AD-like disease.

## Methods

### Animals

Both sexes of Tg2576 mice on a 129S6 genetic background were used (Taconic, Denmark). Tg2576 mice carry a transgene coding for the 695-amino acid isoform of the human AD amyloid precursor protein (APP) and the Swedish mutation (KM670/671NL) [24]. The mice were bred by the provider by backcrossing hemizygous male mice with 129S6/SvEvTac female mice. It is reported that memory deficits in this model start at an age of 6 months and at 9–12 months amyloid plaques in the cortex and hippocampus become apparent similar to Tg2576 mice on a C57BL/6 × SJL genetic background which is a more commonly used strain in the context of AD-like pathology. Mice on 129S6 background, however, are supposed to provide more genetic homogeneity and thus represent a reasonable and validated strain of Tg2576 mice [25]. 129S6 wt mice were used as a control (Taconic, Denmark). Mice were housed at the University of Vienna (Himberg, Austria) and kept under a 12-h light/dark cycle. Food and water was offered ad libitum.

All animal experiments were performed in accordance with the guidelines for care and use of laboratory animals of the Austrian Animal Experiments Act. The protocol was ethical approved and issued by the Lower Austrian Provincial government (permit number: LF1-TVG-22/0102011).

### Peptide vaccine preparation and application

The proprietary AFFITOME® technology [26] was used to develop short immunogenic peptides (AFFITOPE®s), which mimic a C-terminal epitope of murine C5a. This technology enables the design of peptides which induce an antibody response that discriminates between the two forms of C5a, C5a ARG and the metabolized C5a desARG. The peptides (AFFITOPE®s) AFF1 and AFF2 were synthesized by FMOC solid phase peptide synthesis and HPLC-purified (EMC microcollections GmbH, Germany). These peptides contain an additional N-terminal cysteine residue and were activated with the cross linker N-[g-Maleimidobutyryloxy]succinimide ester (GMBS, Pierce) according to manufacturer’s protocol and covalently linked to the carrier Keyhole Limpet Hemocyanin protein (KLH, Biosyn GmbH). The KLH-conjugated peptides were dissolved in 1× PBS (PAA) and absorbed to 0.2 % Alhydrogel® (Brenntag Biosector, Denmark). Thirty micrograms of KLH-conjugated peptides were used for one dose of injection, which was applied subcutaneously in 200 μl. KLH vaccines without the peptide moiety were used as a control vaccine.

### Immunization scheme

Non-AD 129S6 mice (wt mice) were immunized with AFF1- (*n* = 12) and AFF2- (*n* = 12) containing vaccines as well as vehicle control (*n* = 12) at the age of 11 months 3 times in a biweekly interval followed by two further immunizations in monthly intervals.

C5a-peptide vaccination in Tg2576 mice was performed in two studies starting at two different time points. In the first study, which was timely staggered into two parts, Tg2576 mice received either C5a-peptide vaccine AFF1 (*n* = 23) or AFF2 (*n* = 23) at the age of 8 months. All animals were immunized 4 times in a biweekly interval followed by three injections once a month until the age of 13 months. In the second study, the prime immunization of Tg2576 mice was performed at the age of 11 months using only AFF1-formulated vaccine (*n* = 9). These animals were immunized 4 times in a biweekly interval followed by the last immunization a month later. Control treated animals for study 1 (*n* = 14) and study 2 (*n* = 9) were injected with the control vaccine at the same time intervals as the study groups.

### ELISA

To determine the levels of C5a-AFFITOPE® vaccine-induced antibodies, plasma and CSF samples were collected and analyzed by ELISA. Briefly, 1 μM of the antigenic peptides AFF1, AFF2, or an irrelevant control peptide all coupled to BSA were coated in 0.1 M NaHCO_3_ (pH 9.2–9.4) to a 96-well Nunc-MaxiSorp plate. In order to test the reactivity against the target protein, 0.5 μg/ml of the recombinant murine C5a ARG and C5a desARG (Hycult Biotech) were coated in 1× PBS. Free binding sites were blocked by the incubation with blocking buffer (1× PBS, 1 % BSA) for 1 h at 37 °C. Plasma (1:100) and CSF (1:10), respectively, were added, serially diluted 1:2, and incubated for 1 h at 37 °C. For the detection, biotinylated anti-mu IgG (H + L) (Southern Biotech.; 1:2000) in 1× PBS/0.1 % BSA/0.1 % Tween 20 was applied and incubated for 1 h at 37 °C. As a next step, horseradish peroxidase coupled to streptavidin (Roche) was added (30 min, 37 °C) followed by the addition of the substrate 2,2′-Azinobis [3-ethylbenzothiazoline-6-sulfonic acid]-diammonium salt (ABTS) (BioChemica, AppliChem) (30 min, RT). The optical density (OD) at 405 nm was measured with a microwell plate reader (Sunrise, Tecan, Switzerland) and the titers were defined as the dilution factor referring to 50 % of the maximal optical density (OD_max_/2). The mean titers ± SEM of all animals per group are presented.

### Western blot analysis

Two-hundred nanograms of recombinant mouse C5a ARG and C5a desARG (Hycult Biotech) were loaded on a Bio Rad 4–20 % Criterion™ TGX™ gel under non-reducing conditions. For the detection, immune plasma (1:50) of AFF1, AFF2, or control immunized Tg2576 mice as well as plasma obtained from untreated wt mice were used. Rabbit anti-mouse C5a antibody (Abbiotech, 1:200) was used as a control antibody. As a secondary antibody, a goat anti-mouse HRP IgG (1:20.000) for the plasma samples and a rabbit anti-goat HRP IgG (1:20.000) for the control antibody were applied. The Precision Plus Protein™ Dual Color Standards (Bio-Rad) was used as a marker.

### C5a sandwich ELISA

Changes in the C5a levels in the plasma of individual Tg2576 mice before (at the age of 8 months) and after the last immunization (at the age of 15 months) with AFF1-, AFF2-, and vehicle-containing vaccines was determined by a C5a sandwich ELISA. Briefly, 96-well Nunc-MaxiSorp plates were coated with the monoclonal rat anti-mouse C5a antibody (R&D, MAB21501). Plates were blocked with 1× PBS/1 % BSA. Subsequently, plasma obtained 1 day before the first immunization and 8–9 weeks after the last immunization were applied at a starting dilution of 1:10 and serially diluted 1:2 in 1× PBS/0.1 % BSA/0.1 % Tween 20 and incubated for 1 h at 37 °C. For the detection of bound C5a, the biotinylated polyclonal goat anti-mouse complement component C5a antibody (R&D, BAF2150) was applied for 1 h at 37 °C. Horseradish peroxidase coupled to streptavidin (Roche) was added (30 min, 37 °C) followed by the addition of the substrate ABTS (BioChemica, AppliChem) (30 min, RT). The OD at 405 nm was determined by a microwell plate reader (Sunrise, Tecan, Switzerland). The relative changes in OD values at a dilution of 1:40 of plasma samples obtained before (=100 %) and after the last immunization of each individual mouse were evaluated. Plasma obtained from untreated wt mice was used as a control. The group means ± SEM of *n* animals are presented.

### Contextual memory test

Contextual learning and memory of C5a-peptide and control-immunized Tg2576, as well as 129S6 wt mice, were determined by a contextual fear conditioning test at the age of 15 months. A contextual fear conditioning apparatus Ugo Basile 1-cage fear conditioning set-up (UGO BASILE S.r.l. Biological Research Apparatus Via Guido Borghi 43; I–21025 Comerio, Varese; Italy) was used. Fear conditioning training was performed 1 day before the test. Each mouse was placed in the conditioning chamber and exposed to a program allowing the mouse to habituate for 2 min to the chamber, followed by three foot shocks (0.8 mA) in 2-min intervals and a 30-s rest. The movement of the mouse was monitored by a camera (Firewire camera Fire-I BBW 1.3 (Unibrain)) and analyzed by the computerized tracking software ANY-maze (ANY-maze Video Tracking System 4.73–4.91, Stoelting). To assess contextual learning and memory, 24 h after the initial training, the mouse was again placed in the conditioning chamber and the ANY-maze video tracking software was started. This time, the animal received no electric shocks. Each mouse was monitored in the chamber for 5 min for immobile episodes (i.e., time freezing) representing their ability to recall the foot shocks they received the previous day.

### Modified SHIRPA test

129S6 wt mice were immunized with AFF1-, AFF2-, or vehicle-containing vaccines starting at the age of 11 months and at the age of 15 months, comprehensive analyses of general health, muscle function, and sensory function were performed by a modified SHIRPA test as previously described at the European Mouse Phenotyping Resource Standardized Screens (EMPReSS) database (http://empress.har.mrc.ac.uk/). Briefly, eye closure (score 0 = no, score 1 = yes/normal), missing whiskers (score 0 = no/normal, score 1 = yes), tail curl (score 0 = abnormal, score 1 = normal), respiratory function (score 0 = gasping/arrhythmic, score 1 = normal), and body weight were evaluated for general health parameters. For the evaluation of the sensory abilities, visual forepaw reach (score 0 = no, score 1 = weak, score 2 = clear placing reaction/normal), auditory startle (score 0 = no, score 1 = present/normal), and ear and whisker twitch (score 0 = absent, score 1 = present/normal) were assessed. The muscle functions were evaluated based on pelvic elevation (score 0 = low, score 1 = normal), truck curl (score 0 = absent, score 1 = present/normal), righting reflex (score 0 = absent/impaired, score 1 = present/normal), clasping (score 0 = no, score 1 = 1 paw, score 2 = 2 paws), forelimb grip (score 0 = no, score 1 = weak, score 2 = normal), and grasping reflex (score 0 = no/all 4 paws impaired, score 1 = fore and hind limbs impaired, score 2 = all 4 paws normal).

### Immunohistochemistry and image analysis

The mouse brain was isolated and without perfusion, the left hemi-brain was fixed o/n in 4 % paraformaldehyde (PFA, Merck) at 4 °C. The tissue was embedded in paraffin, and coronal sections (7 μm) containing the frontal, middle, and distal hippocampal region (bregma distance −1.60, −2.10, and −2.60 mm) were used for immunohistochemical staining. After rehydration and treatment with antigen retrieval pH = 9.0 (Dako), the tissues were blocked with 4 % normal goat or rabbit serum, respectively (Sigma). Serial sections were incubated with the corresponding primary antibody or control IgG. Aβ deposits were detected by an in-house generated mouse 3A5 monoclonal antibody (mAB) that specifically binds to the N-terminal part of Aβ [27]. Activated CD45^high^ microglia cells were visualized by the goat anti-mouse CD45 antibody (AF114) obtained from R&D systems. Fluorescent anti-mouse IgG (H + L) and Fluorescent anti-goat IgG (H + L) (Vector Laboratories) were used for the detection of 3A5 and CD45 antibody, respectively. The cover slides were mounted with Vectashield® Hard Set™ Mounting Medium with 4′,6-diamidino-2-phenylindole (DAPI) (Vector Laboratories), dried for 2 days in the dark. Slides were then scanned by the Mirax Scan 150 automated whole slide scanner (Karl Zeiss AG). The relative amyloid plaque area (3A5 staining) within the total tissue area of coronal cross sections of the brain including the cortex, hippocampus, and the brain stem was analyzed by a semi-automated area recognition program (eDefiniens Architect XD; www.definiens.com). CD45^high^ cells in the hippocampal region were counted manually. Immunostaining and analysis of each marker was performed from 6–12 sections of each individual mouse brain derived from 3 defined bregma distances all including the hippocampal region. The mean value of all sections per mouse was calculated and the means per group ± SEM of *n* animals are presented.

### Statistical analysis

All values were evaluated for homoscedasticity and normality assumption using both Kruskal-Wallis and Shapiro-Wilk normality tests. To determine statistical significance of more than two groups, values were compared using one-way ANOVA followed by the Tukey’s multiple comparison tests (parametric test) or one-way ANOVA with the Dunn’s test (non-parametric test). For the comparison of two groups, the unpaired two-tailed Student’s *t*-test was used, followed by the Mann-Whitney correction for non-parametric data as indicated in the respective figure legend.

The *p* values ≤0.05 were considered significant and are expressed as ^*^p < 0.05 and ^**^p < 0.01.

## Results

### Immunogenicity and safety of anti-C5a-peptide vaccines in wt mice

**Fig. 1:**
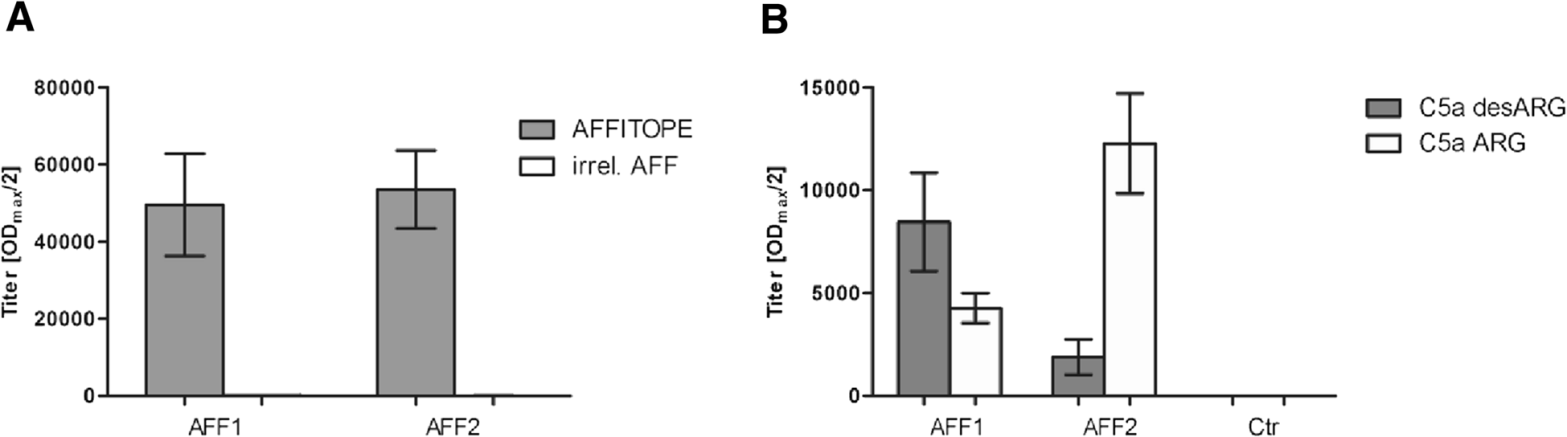
C5a-peptide vaccines are immunogenic and induce a target-specific antibody response in wt mice. 129S6 wt mice were immunized 4 times with AFF1- (*n* = 12) and AFF2- (*n* = 12) containing vaccines starting at the age of 11 months. Control mice (*n* = 12) were immunized 4 times with a KLH-only vaccine. Plasma samples obtained after the last immunization were analyzed by ELISA. **a** The mean IgG antibody titers (OD_max_/2) against the injected peptides AFF1 and AFF2 and against an irrelevant peptide. **b** The mean titers against the target protein C5a present in its two forms C5a desARG and C5a ARG. Bars represent the group means ± SEM of n animals

Peptides (AFFITOPE®s) mimicking the C-terminal neo-epitope of murine C5a were designed, formulated, and tested in 129S6 wt mice for their immunogenicity and ability to induce antibodies against the endogenous target protein C5a (Fig. 1). C5a is present in two different forms, the highly active C5a ARG which becomes rapidly metabolized by a carboxypeptidase to the less active though more stable C5a molecule without the C-terminal arginine, C5a desARG. As the relevance of the two different forms of C5a in neuro-inflammatory disease is still unknown, different C5a-targeting peptides (AFFITOPE®s), AFF1 and AFF2, have been selected for in vivo testing. Both AFF1- and AFF2-containing vaccines elicited similarly high titers of approximately 1/50.000 against their antigenic peptide moiety (Fig. 1a). No cross-reactivity against an unrelated irrelevant peptide was observed (Fig. 1a). However, their reactivity against the different forms of the target protein differed. AFF1-containing vaccine mounted higher antibody titers against C5a desARG, whereas AFF2-containing vaccine primarily induced a strong immune response against the more active molecule, C5a ARG (Fig. 1b). As expected, control immunized mice did not show any immune response against C5a ARG and C5a desARG (Fig. 1b).

**Fig. 2:**
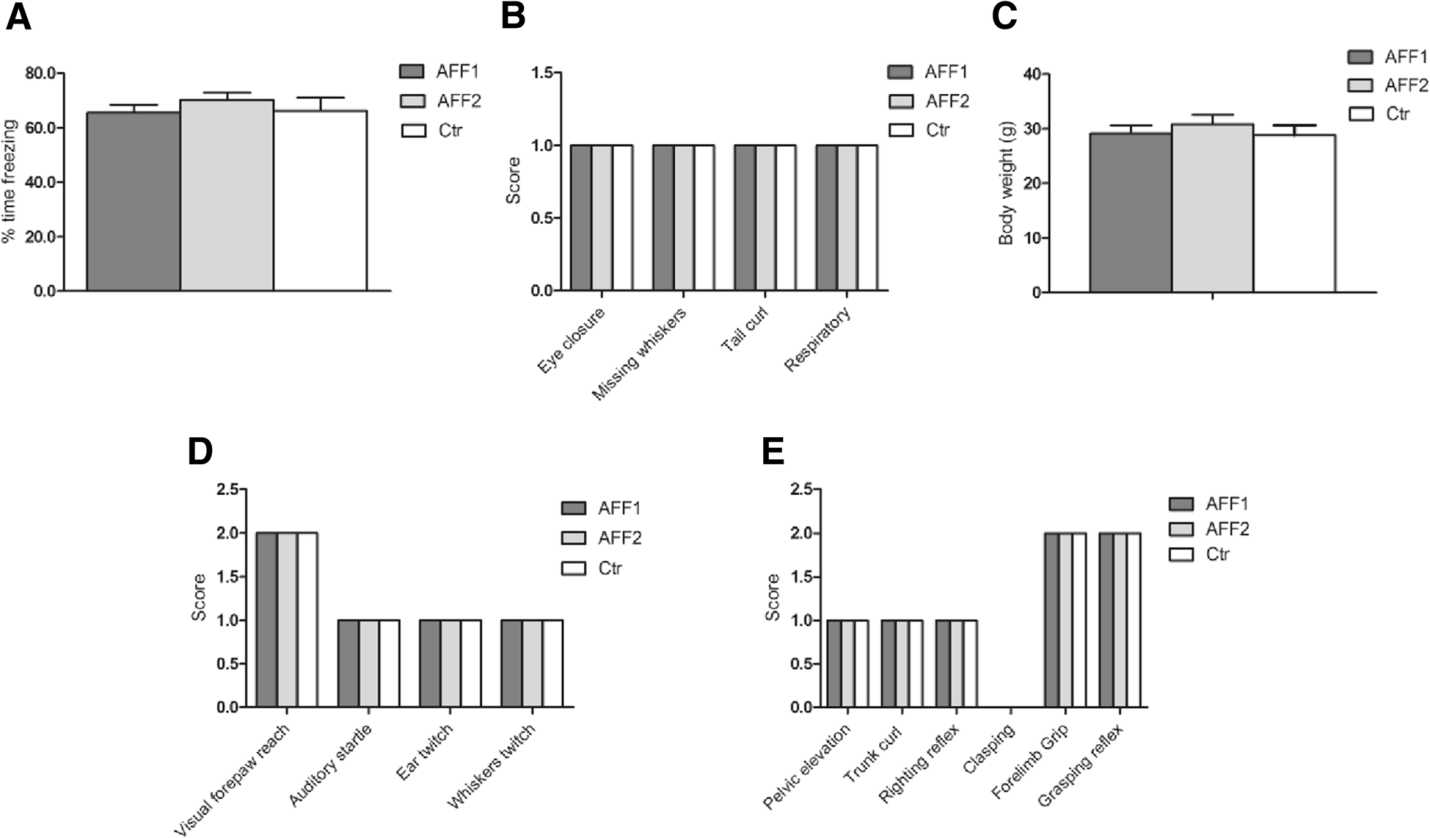
Wt mice treated with C5a-peptide vaccines show normal contextual learning and memory skills and are healthy with normal motor and sensor function. 129S6 wt mice immunized with AFF1- (*n* = 12) and AFF2- (*n* = 12) containing vaccines were compared to vehicle immunized mice (*n* = 12) for **a** contextual memory function using a contextual fear conditioning test, **b** general health status, **c** body weight, **d** sensory functions, and **e** motor functions using the modified SHIRPA test. All tests were performed at the age of 15 months. *Bars* represent the group means ± SEM of *n* animals

Before testing the C5a-peptide vaccines for their efficacy in a mouse model of AD, we investigated whether these vaccines had any effect on the contextual learning and memory in 129S6 wt mice by using the contextual fear conditioning test. After four immunizations, no differences between the control and the C5a-AFFITOPE® immunized mice were observed all showing around 60 % time freezing within a 2-min period of analysis (Fig. 2a). Furthermore, potential side effects of the vaccines were evaluated by a modified SHIRPA test including general health, muscle, and sensory functions. C5a-peptide and vehicle-immunized 129S6 wt mice were all healthy (Fig. 2b), had comparable body weight (Fig. 2c), and no abnormalities in sensory and motor functions were observed (Fig. 2d, e). In addition, different safety parameters were elucidated by in silico analyses. The absence of target-specific T cell response and cross-reactivity of vaccine-induced antibodies to other endogenous proteins are a prerequisite for an immunotherapy against self-proteins in order to avoid the risk of autoimmunity. In silico analysis by online available T cell epitope prediction algorithms (e.g., http://www.syfpeithi.de or http://www.iedb.org/counts.php) did not predict any relevant T cell epitope for the peptides AFF1 and AFF2. Potential cross-reactivity of the AFF1- and AFF2-induced antibodies to other proteins was evaluated by BLAST searches against the murine proteome. AFF1 did not show any relevant homology to other murine proteins. For AFF2, a homology of 6 amino acids to FYN binding protein was found, however, based on its intracellular location, it is not anticipated to be accessible for antibody binding.

### Immunogenicity in Tg2576 mice

**Fig. 3:**
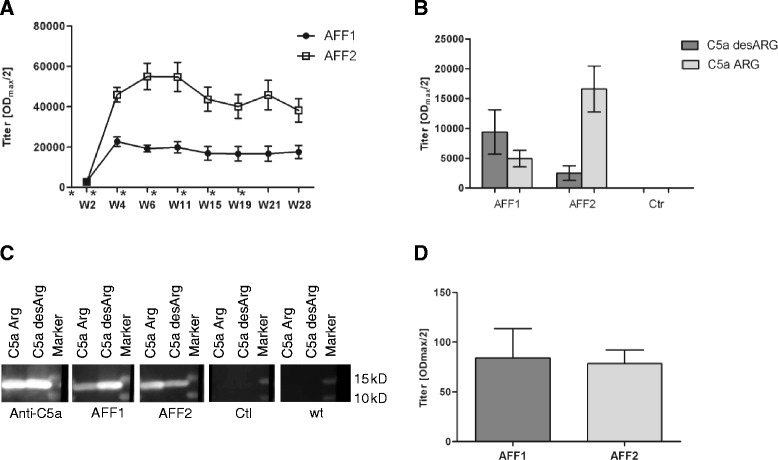
C5a-peptide vaccines induce target-specific antibody response in Tg2576 mice. **a** Tg2576 mice were immunized with AFF1- (*n* = 12) and AFF2- (*n* = 13) containing vaccines 4 times in biweekly intervals (week 0, 2, 4, and 6) followed by 3 boosts in a monthly interval (week 11, 15, and 19). The immunizations are indicated by an asterisk. Plasma samples were collected at the indicated time points (week 2–28). All samples were analyzed by ELISA and the mean IgG antibody titers (OD_max_/2) against the injected peptide AFF1 and AFF2 are presented. **b** The titers against both forms of the target protein, C5a desARG and C5a ARG, were analyzed in the plasma obtained after the last immunization in W28. Also control immunized mice (*n* = 11) were tested against C5a proteins (**c**). Western blot analyses where recombinant C5a ARG and C5a desARG were loaded and detected by AFF1 (*second panel*), AFF2 induced immune plasma (*third panel*), as well as plasma obtained from control immunized mice (*fourth panel*), and untreated wt mice (*fifth panel*) as a negative control. Rabbit anti-mouse C5a antibody was used as a positive control (*first panel*). The Precision Plus Protein™ Dual Color Standards (Bio-Rad) was used as a marker. **d** CSF was obtained from five randomly selected AFF1 and AFF2 immunized mice and tested for AFFITOPE®-specific antibodies in week 28. All data points represent the group means ± SEM of *n* animals

Human APP transgenic Tg2576 mice on a 129S6 genetic background were used as a model of AD-like disease. In a first study, starting at the age of 8 months, Tg2576 mice were either immunized with AFF1- or AFF2-formulated anti-C5a vaccines. Two weeks after the second immunization (W4), titers against the immunizing peptides AFF1 and AFF2 approached approximately 1/20.000 and 1/45.000, respectively, and remained at this level until week 28 (W28) corresponding to 15 months of age (Fig. 3a). Compared to the results obtained from immunized wt mice, AFF2-containing vaccines induced similar high titers against the antigenic peptide AFF2, whereas AFF1-containing vaccines elicited a clearly lower titer against the peptide moiety (Figs. 1a and 3a). ELISA for the proteins C5a desARG and C5a ARG revealed that AFF1-containing vaccine mounted higher antibody titers against C5a desARG whereas AFF2-containing vaccine showed a much higher immune response against C5a ARG (Fig. 3b), similar to those results obtained from AFF1 and AFF2 immunized wt mice (Fig. 1b). As expected, control immunized mice did not show any immune response against the C5a proteins (Fig. 3b). Western blot analyses using recombinant C5a ARG and C5a desARG confirmed this reactivity pattern of AFF1- and AFF2-induced antibodies. Again, AFF1-elicited antibodies showed a better recognition for C5a desARG, whereas AFF2-induced immune plasma revealed a stronger band for C5a ARG. Control immunized mice as well as plasma obtained from untreated wt littermates at the age of 15 months did not show any signal toward both forms of C5a (Fig. 3c).

In order to assess whether AFF1- and AFF2-induced antibodies reach their target in the brain, cerebrospinal fluid (CSF) was obtained and analyzed for the presence of AFFITOPE®-specific antibodies by ELISA. Antibodies could be detected in the CSF ranging from 0.2 to 0.4 % of their corresponding titers in plasma (Fig. 3d).

### C5a-peptide vaccines deplete C5a from the circulation

**Fig. 4:**
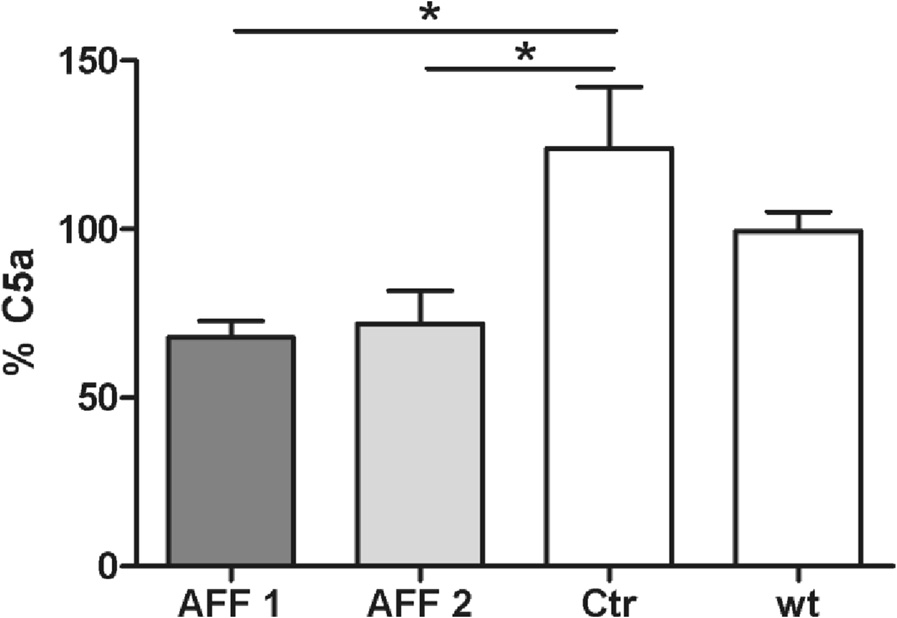
Depletion of C5a from the circulation through C5a-peptide vaccination. The changes of C5a concentration in the plasma of Tg2576 mice before (at the age of 8 months) and after the treatment (at the age of 15 months) with AFF1- (*n* = 12), AFF2- (*n* = 13), or vehicle- (*n* = 11) containing vaccines were determined by sandwich ELISA. The level of C5a in the plasma obtained before the immunization start at the age of 8 months refers to 100 %. The C5a level in the plasma of untreated wt mice (*n* = 6) at the age of 8 months vs. 15 months is also shown. *Bars* represent the group means ± SEM of *n* animals. The one-way ANOVA test followed by a Tukey’s multiple comparison Test (parametric test) was applied to determine the *p* values (^*^ *p* < 0.05 and ^**^ *p* < 0.01)

The plasma concentration of C5a was used as a surrogate for antibody binding efficacy to C5a of vaccine-induced antibodies. The changes of the total C5a levels in the plasma obtained before (refers to 100 %) and after the last immunization of AFF1, AFF2, and control immunized Tg2576 mice were assessed. The levels of C5a were significantly reduced to 68 and 72 % in AFF1- and AFF2-treated mice, respectively, whereas control immunized mice showed an increase of C5a up to 124 % (Fig. 4), indicating an in vivo target engagement of AFFITOPE®-induced antibodies. As an additional control, plasma from untreated wt mice was analyzed showing a constant level of C5a in the plasma of 8- vs. 15-month-old mice (Fig. 4).

### Early stage immunization improves contextual memory and reduces the number of activated microglia and amyloid plaque load in Tg2576 mice

**Fig. 5:**
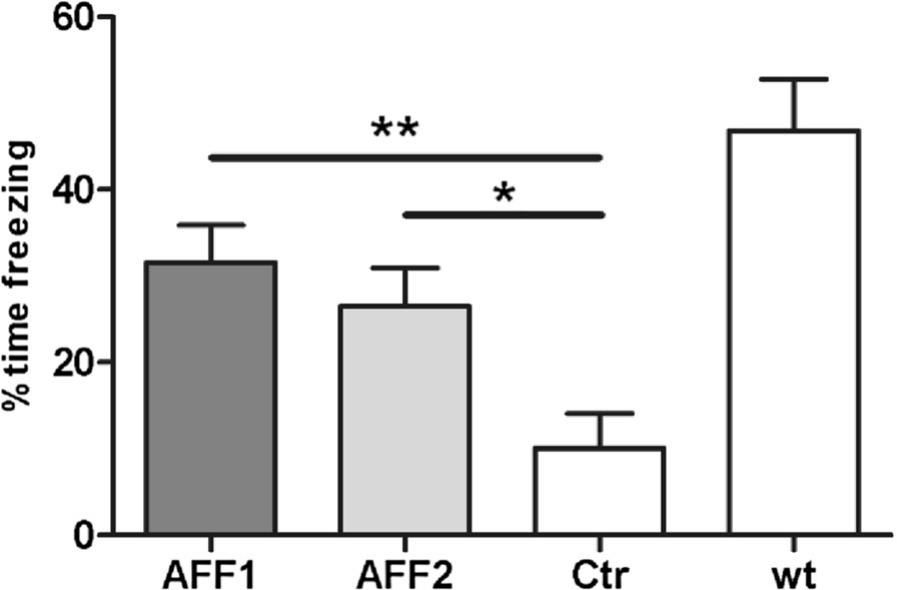
Contextual memory is significantly improved following C5a-peptide vaccination. A CFC test was performed with AFF1 (*n* = 23), AFF2 (*n* = 23), and control (*n* = 14) immunized Tg2576 mice, as well as wt littermates (*n* = 12), all at the age of 15 months. Mice were exposed to electric foot shocks and the memory to recall these shocks was assessed by the percent time freezing in a 2-min interval. *Bars* represent the group means ± SEM of *n* animals. The *p* value was determined using one-way ANOVA test followed by a Dunn’s multiple comparison Test (non-parametric test). The *p* values are expressed as ^*^ *p* < 0.05 and ^**^ *p* < 0.01

In a first set of experiments, anti-C5a immunization of Tg2576 mice by AFF1- and AFF2-containing vaccines was started at the age of 8 months, where first memory deficits are reported to become apparent in this model, but amyloid plaques in the brain are not yet present. A contextual fear conditioning test was performed at the age of 15 months in order to investigate the impact of C5a-targeting vaccines on the progression of AD-like cognitive decline. The time freezing was used to measure mice’s ability to recall the shocks they received on the previous day. Both AFF1- and AFF2-vaccinated mice showed significantly increased memory retention with 32 and 27 % time freezing, respectively, whereas control-treated mice spent only 10 % time freezing indicating an almost complete loss of contextual learning and memory (Fig. 5). However, vaccination was not able to restore the contextual learning and memory conditions which were found in untreated wt littermates (47 % time freezing) (Fig. 5). Non-AD wt littermates have not been included in this study. To evaluate that the memory improvements in AFF1- and AFF2-vaccinated Tg2576 mice can be attributed to the neutralization of excessive C5a which was provoked by the APP transgene, an independent control experiment was performed. Wt mice were immunized 4 times with AFF1 and AFF2 vaccines and tested for memory retention by a contextual fear conditioning test. No differences between the control and the C5a-AFFITOPE®-immunized mice were found as already shown in Fig. 2a.

**Fig. 6:**
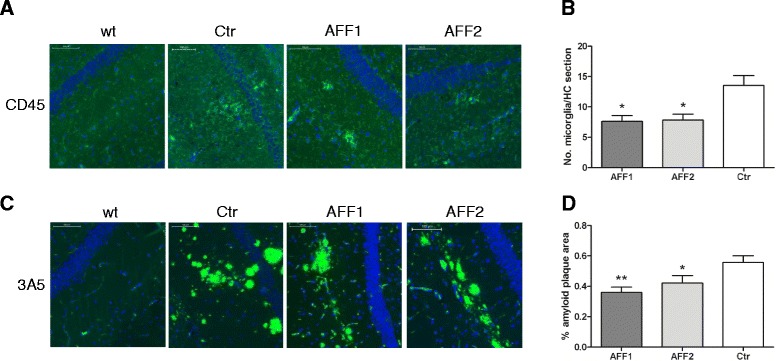
At an early stage of disease, C5a-peptide vaccines reduce the number of activated microglia and amyloid plaque load in the brain. **a** Brain sections of 15-month-old Tg2576 mice immunized with either AFF1 (*n* = 23), AFF2 (*n* = 23), or control (*n* = 14) vaccines were stained for CD45-positive microglia cells (*green*). **b** The average number of CD45^high^ cells in the hippocampal region of all sections. **c** Amyloid plaques visualized by the 3A5 mAB (*green*) (**d**). Percentage of amyloid area of the total brain sections all containing the hippocampal region was calculated by the eDefiniens Software. **a**, **c** Cell nuclei were stained with DAPI (*blue*) and the *scale bar* refers to 100 μm. **b**, **d** *Bars* represent the group means ± SEM of *n* animals. The *p* value was determined using one-way ANOVA test followed by a Dunn’s multiple comparison Test (non-parametric test) and expressed as ^*^ *p* < 0.05 and ^**^ *p* < 0.01

In order to investigate the impact of anti-C5a vaccination on microgliosis in the hippocampus, immunohistochemical analysis of CD45^high^ cells in the brain of AFF1-, AFF2-, and control-treated Tg2576 mice at the age of 15 months was performed (Fig 6a). Compared to control, both AFF1- and AFF2-formulated vaccines significantly reduced the number of CD45-positive (CD45^high^) microglia in the hippocampus (Fig. 6b).

We further analyzed whether anti-C5a vaccination also had an influence on the cerebral Aβ pathology, a major hallmark of AD progression. The in-house generated Aβ-specific mAB 3A5 [27] was used to detect Aβ depositions in the brain of 15-month-old mice treated with AFF1, AFF2, or the control vaccine (Fig. 6c). Both, AFF1- and AFF2-treated mice, revealed a statistically significant reduction of cerebral amyloid burden exhibiting 0.39 % in AFF1 (*p* < 0.01) and 0.42 % (*p* < 0.05) in AFF2 amyloid plaque area of the total coronal brain section, compared to 0.55 % in control immunized mice (Fig. 6d).

### Late-stage vaccination improves memory function and reduces the number of activated microglia but has no influence on amyloid burden in the brain

**Fig. 7:**
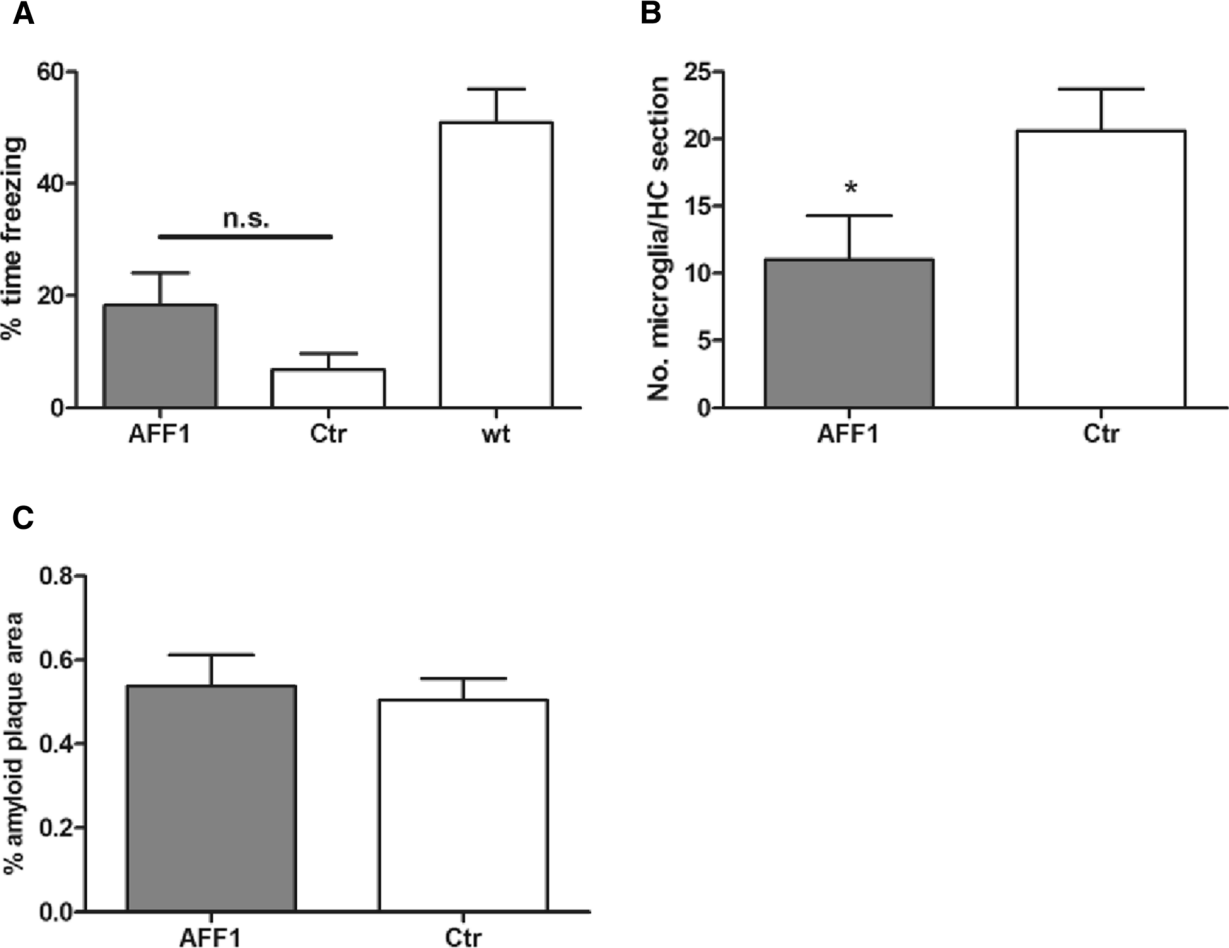
Vaccination at a progressed stage of disease improves memory function and reduces microgliosis but had no effect on the amyloid plaque load in the brain. **a** Tg2576 mice which were immunized with AFF1- (*n* = 9) and control (*n* = 9) vaccines at the age of 11 months, as well as wt littermates (*n* = 24) were exposed to a contextual fear conditioning test. **b** Average number of CD45^high^ cells in the hippocampal region analyzed by immunohistochemistry. **c** The percentage of amyloid plaque area of the total brain sections. The *bars* represent the group means ± SEM of *n* animals. The unpaired two-tailed Student’s *t*-test was used for statistical analysis, whereas in Fig. 7a, a Mann Whitney correction for non-parametric data was performed. A *p* value of ≤0.05 was considered to be statistically significant

In order to determine if the beneficial effects of our anti-C5a vaccines which were observed upon early stage treatment could also be seen in a more progressed stage of Alzheimer-like disease, AFFITOPE® immunization was started at the age of 11 months, where memory deficits and cerebral amyloid depositions are already clearly pronounced. Mice were immunized with AFF1-containing vaccines 4 times in a biweekly interval followed by the final immunization 1 month later. A contextual fear conditioning test was performed at the age of 15 months in order to assess the influence of the anti-C5a vaccination on memory impairment. Compared to the control vaccine, AFF1-vaccinated mice showed a tendency of better contextual learning and memory skills indicated by 18 % vs. 7 % time freezing (Fig. 7a**,***p* = 0.182). Untreated wt littermates showed 51 % time freezing similar to the result obtained in Fig. 5. Again, wt littermates treated with AFF1 vaccine have not been included in this study. However, an independently performed control experiment showed that the memory retention of control and AFF1-immunized wt mice was similar (see Fig. 2a).

When evaluating the number of CD45^high^ microglia cells in the hippocampus, a significant reduction in AFF1 compared to control-treated Tg2576 mice was found (Fig. 7b).

In contrast to Tg2576 mice immunized at an early stage of the disease (Fig. 6d), cerebral amyloid burden was not reduced upon late-stage treatment with anti-C5a vaccine (Fig. 7c).

## Conclusion

Active vaccination against the pro-inflammatory molecule C5a represents a novel and well-tolerated therapeutic approach to interfere with neuroinflammation in a mouse model of AD. Early- as well as late-stage treatment with C5a-peptide vaccines reduced microgliosis and improved cognitive function. Amyloid plaque burden, however, was only affected in an early onset of vaccination, thus we suppose that C5a-mediated neuroinflammation more than amyloid β aggregation is a driving factor for memory decline in this model of AD.

## Discussion

The present study shows that active immunization with C5a-peptide vaccines AFF1 and AFF2 elicits antibodies that effectively bind C5a and reduce the number of activated microglia in the hippocampus, accompanied by memory improvements in a mouse model of AD. Moreover, cerebral amyloid plaque load was decreased upon an early-stage treatment with C5a-peptide vaccines.

An early characteristic of AD is the generation of amyloid proteins by the increased proteolytic cleavage of amyloid precursor proteins (APP) which start to accumulate and aggregate especially in the hippocampal and cortical region of the brain. Neuroinflammation also plays a critical role in the pathogenesis of AD, although the mechanisms through which amyloid aggregation and deposition provoke inflammation are not fully understood. Microglia attraction and activation by amyloid plaque formation may play a detrimental role (reviewed in [10, 12]). Microglia can be activated by direct interaction with misfolded or aberrant endogenous molecule patterns via pattern recognition receptors (PRRs) including scavenger receptor (SR-AI/II), CD36, RAGE, FC receptor, and toll-like receptors (TLRs) [28, 29]. Another important mechanism of microglia recruitment and activation is complement activation in response to Aβ deposition [30, 31] which leads to the formation of the pro-inflammatory molecule C5a. C5aR was shown to be up-regulated in microglia in the immediate surroundings of plaques in the brain of different transgenic mouse models of AD [20].

**Fig. 8:**
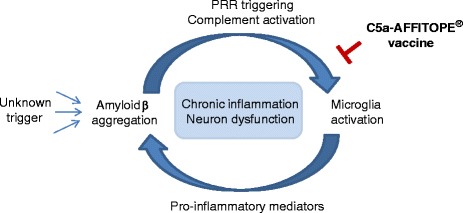
Model of C5a-peptide vaccines interference with chronic, self-sustaining inflammatory processes in AD. APP processing to amyloid peptides (Aβ 1–42) is influenced by an unknown trigger. Aβ peptides start to aggregate in the brain and microglia become activated either by a direct interaction with misfolded Aβ molecules via pattern recognition receptors (PRR) or by complement activation in the response to Aβ depositions leading to the formation of the pro-inflammatory molecule C5a. We propose that C5a-AFFITOPE® vaccines interfere with the pro-inflammatory molecule C5a thereby hindering enhanced microglia activation which leads to excessive release of pro-inflammatory mediators. These pro-inflammatory mediators provoke stress conditions, which in turn reinforce APP production and processing to amyloid peptides (Aβ 1–42). We believe that C5a-AFFITOPE® vaccines do have the capacity to interfere with chronic and self-sustaining inflammatory interactions between activated microglia, stressed neurons, and Aβ plaques which ultimately lead to neuronal cell death and cognitive decline in AD patients

Activation of microglia by Aβ and complement is supposed to promote the excessive release of pro-inflammatory cytokines [32], chemokines [33, 34], and further complement components [35, 36], as well as the release of reactive oxygen and nitrogen species [37, 38], altogether leading to dysfunction and loss of synapse signaling [39]. Pro-inflammatory mediators provoke a number of stress conditions which, in turn, can enhance APP production and processing to amyloid peptides (Aβ 1–42) [40–45]. Thus, chronic and self-sustaining inflammatory interactions between the complement system, activated microglia, stressed neurons, and Aβ plaques occur, which ultimately lead to neuronal cell death and cognitive decline in AD patients. Our intention was to target the pro-inflammatory complement activation product C5a in order to interfere with enhanced microglia activation and sustained neuroinflammation in AD (Fig. 8).

There are several publications that describe a physiological role for the anaphylatoxins C3a and C5a within the adult murine CNS, specifically an involvement in cell survival and neurogenesis ([46] and reviewed in [47–49]). Neuroprotective effects of C3a and C5a have been reported against glutamate-mediated neuronal excitotoxicity via MAPK-dependent inhibition of caspase-3 [50–52] and increased glial expression of the glutamate transporter GLT-1 [53]. In addition, administration of C5a in vivo has been shown to protect against kainic acid-induced neuronal apoptosis [52]. However, activation of complement and the release of C3a and C5a to an inappropriate extent has been proposed to promote tissue injury.

In the pathogenesis of AD, the complement factors C1q [54] and C3 [55] have been reported to promote phagocytosis and clearance of fibrillar Aβ and thus hinder pathologic progression in AD. The complement component C5a, however, is supposed to play a detrimental role in neurodegenerative diseases by persistent microglia activation resulting in excessive release of microglial inflammatory mediators, which enhance Aβ deposition and neuroinflammation [40, 42, 43]. It was shown that the blockage of C5aR by the antagonist PMX205 lead to a therapeutic benefit in different models of neurodegeneration [21, 22], which strengthens our hypothesis that interference with C5a by vaccination ameliorates AD pathology. The antigenic peptides AFF1 and AFF2, when coupled to the carrier protein KLH and formulated with Alhydrogel®, were able to induce a strong and prolonged humoral immune response against the target protein C5a in mice. Upon immunization, a reduction of total C5a in the plasma was found which indicated an effective in vivo target engagement. AFF1 and AFF2 were selected for in vivo studies, because these two peptides possess different immunogenic features. AFF1-induced antibodies react against both forms of C5a, the highly active C5a ARG but to a higher extent against the metabolized, less active, and more stable C5a desARG (Figs. 1b and 3b), whereas immune plasma elicited by AFF2 vaccines especially showed high titers against C5a ARG (Figs. 1b and 3b). To date, the distinct function of the two forms of the anaphylatoxin C5a and their contribution to chronic inflammatory diseases, such as AD, are not understood. AFF1 compared to AFF2 vaccine was found to be slightly more efficient in Tg2576 mice in terms of contextual memory retention (Fig. 5) raising the possibility that C5a desARG more than C5a ARG contributes to neuro-inflammation in AD.

Most of the vaccine-induced antibodies were present in the circulation, however, a lower proportion (0.2–0.4 %) can also be detected in the CSF which let us assume that vaccine-induced anti-C5a antibodies directly reach their target in the brain. Immunohistochemistry, ELISA, and Western blot analyses were performed in order to show local anti-C5a antibody effects, but unfortunately the sensitivity of all these methods was not sufficient to detect C5a in the brain. However, besides local anti-C5a antibody effects, the clearance of C5a from the periphery, which we were able to show in Fig. 4, may play a crucial role in the treatment of neurodegenerative diseases, since a secondary systemic inflammatory stimulus is hypothesized to affect exaggerated response of microglia which possibly contributes to disease progression (reviewed in [56, 57]).

We found that both early (at the age of 8 months) and late (at the age of 11 months) onset of anti-C5a immunotherapy reduced the number of activated microglia in the hippocampus (Figs. 6b and 7b) and improved contextual learning and memory in 15-month-old Tg2576 compared to control-treated mice (Figs. 5 and 7a) indicating that neuroinflammation is directly involved in memory decline and C5a may be a crucial factor that drives this inflammation. To be sure that amyloid is the main factor that provokes complement activation in Tg2576 mice and thus memory decline, an additional group of wt littermate mice immunized with anti-C5a vaccines should have been included in the above presented experiments. Since these data are not available, we cannot exclude that a secondary inflammatory stimulus may contribute to the outcome or progression of AD-like disease in these animals. However, the effect of anti-C5a immunization on non-transgenic mice was tested in an independently performed control experiment which showed that C5a-AFFITOPE® immunization did not influence the cognitive behavior (Fig. 2a).

Besides the contextual fear conditioning test, also the classical Morris water maze test, another hippocampal dependent complex learning tasks, was performed. Similar results were obtained (data not shown), although a contextual fear conditioning test seems to be more reliable. As analyzed by Owen et al., contextual fear conditioning detected less variability across genotypes and thus appears to be more broadly applicable to studies of complex learning in mice compared to Morris water maze task [58].

Cerebral amyloid plaque load was only reduced in mice vaccinated at an early and not at a late stage of Alzheimer-like disease (Figs. 6d and 7c). This observation indicates that already existing amyloid β plaques cannot be reduced through anti-C5a vaccination whereas initial deposition can be alleviated significantly by anti-C5a interference. Interestingly, Fonseca et al. found a reduction of amyloid plaque load in Tg2576 mice upon treatment with PMX205, a C5aR inhibitor, starting at the age of 12 months [22]. The difference between our study and previously presented data may be due to the different interference strategies. Specifically, treatment with small molecules, such as PMX205, may immediately be effective, whereas an active immunization approach using C5a-peptide vaccines takes at least 4–6 weeks to mount an appropriate antibody response.

The major advantage of an active immunotherapy vs. the application mAbs or small molecules however is that prolonged therapeutic effects can be easily achieved by booster immunizations (Fig. 3a) whereas mAB and small molecule therapy is linked to frequent treatments and mostly high costs.

All immunotherapeutic approaches which target self-proteins have to consider the issue of potential autoimmunity. Thus, a prerequisite of our peptide vaccines was to stimulate the humoral immune system and induce specific and effective antibodies against complement factor C5a without inducing target-specific T cells and thereby avoiding a potential autoimmune response with life-threatening consequences. The proprietary AFFITOME® technology favors the use of short epitope-specific peptides (<13 aa) which do not bind to MHC class II molecules. Peptides (AFFITOPE®s) become antigenic only when coupled to a carrier protein (e.g., KLH) which provides T helper cell epitopes. Thus, immunization with AFF1 and AFF2 induces an effective B cell response and concurrently circumvents target-specific CD4 T cell activation. However, peptides which exceed 7 amino acids potentially activate MHC class I-restricted CD8 positive T cells. Analysis by online available T cell epitope prediction algorithms did not predict any relevant T cell epitope for the anti-C5a peptides AFF1 and AFF2. These in silico data were further confirmed by in vivo immunization experiments where no abnormalities in terms of mortality, weight loss, sensory and motor functions, handling behavior, and contextual memory skills of wt mice treated with the C5a-peptide vaccines were observed (Fig. 2a–d). Therefore, C5a-peptide vaccines represent a safe and well-tolerated immunotherapy, which is able to elicit a specific and long lasting immune response against C5a (Figs. 1a and 3a) without any relevant cross-reactivity. Another safety aspect of our vaccination approach was to target only C5a and not C5 or C5b, in order to interfere only with a terminal detrimental component of the complement system. Potentially protective complement activation events more upstream important for the clearance of aggregated proteins and cell debris will be maintained [54].

We showed that C5a-AFFITOPE® vaccination is able to target the pro-inflammatory complement activation product C5a, thus preventing enhanced microglia activation in the hippocampus, reducing memory decline and Aβ pathology in a model of AD.

Thus, active immunotherapy against complement factor C5a is a new and effective approach for the treatment of AD-like disease. Moreover, these promising results suggest a therapeutic potential of C5a vaccination not only in AD but also for other indications in which chronic inflammatory processes driven by complement activation may play a pivotal role.
